# Genomic resources of broomcorn millet: demonstration and application of a high-throughput BAC mapping pipeline

**DOI:** 10.1186/s12863-021-01003-z

**Published:** 2021-11-01

**Authors:** Wei Xu, Mengjie Liang, Xue Yang, Hao Wang, Meizhong Luo

**Affiliations:** College of Life Science and Technology, Huazhong Agricultural University, Wuhan, 430070 China

**Keywords:** Broomcorn millet, BAC, BES, Genomics resources, Gap filling, CAPSS, Jbrowse

## Abstract

**Background:**

With high-efficient water-use and drought tolerance, broomcorn millet has emerged as a candidate for food security. To promote its research process for molecular breeding and functional research, a comprehensive genome resource is of great importance.

**Results:**

Herein, we constructed a BAC library for broomcorn millet, generated BAC end sequences based on the clone-array pooled shotgun sequencing strategy and Illumina sequencing technology, and integrated BAC clones into genome by a novel pipeline for BAC end profiling. The BAC library consisted of 76,023 clones with an average insert length of 123.48 Kb, covering about 9.9-fold of the 850 Mb genome. Of 9216 clones tested using our pipeline, 8262 clones were mapped on the broomcorn millet cultivar longmi4 genome. These mapped clones covered 308 of the 829 gaps left by the genome. To our knowledge, this is the only BAC resource for broomcorn millet.

**Conclusions:**

We constructed a high-quality BAC libraray for broomcorn millet and designed a novel pipeline for BAC end profiling. BAC clones can be browsed and obtained from our website (http://eightstarsbio.com/gresource/JBrowse-1.16.5/index.html). The high-quality BAC clones mapped on genome in this study will provide a powerful genomic resource for genome gap filling, complex segment sequencing, FISH, functional research and genetic engineering of broomcorn millet.

**Supplementary Information:**

The online version contains supplementary material available at 10.1186/s12863-021-01003-z.

## Background

With the increased global water scarcity caused by climate change and population growth, it is of great importance to exploit the high-efficient water-use crop for food security of the human beings in the future. Broomcorn millet (*Panicum miliaceum*), also known as proso millet, panic millet and wild millet, is one of the traditional five-grain crops in the north of China [1]. It is a typical C_4_ plant with high photosynthetic efficiency. It has also a high-efficient utilization ratio of water resource and a capacity for drought resistance, exquisitely adapting to semi-drought or drought conditions [2]. Furthermore, its growing cycle, 60–90 days from sowing to maturity, is shorter than other cereals [3]. Broomcorn millet contains more protein than most grains, and a relatively balance array of trace elements and vitamins [4]. More than 8700 accessions of broomcorn millet including landraces and cultivars have been conserved in the National Gene Bank of the Institute of Crop Science, Chinese Academy of Agricultural Sciences, thus providing an abundance of resources for genetic improvement of broomcorn millet.

The gold-standard reference genomes for understanding genetic relationships among germplasm resources is extremely overaching [5]. It can facilitate the comprehensive exploration to thoroughly find out the quantitative trait locus (QTL) with speical characters like resistance, yield and quality [6, 7]. At present, high-quality chromosome-scale genome assemblies of two allotetraploid (2n = 4x = 36) broomcorn millet varieties decoded by Chinese researchers are available [8, 9]. These genome assemblies provide the foundation for the molecular breeding of broomcorn millet. However, other genomic resources are still required to complete and make the full use of the genome assemblies.

The tranditional bacterial artificial chromosome (BAC) libraries with genomic DNA inserts of 50 kbp – 300 kbp [10–12] are also important resources for genomic research. In the past two decades, hundreds of robust BAC libraries and corresponding physical maps have been constructed for many vertebrates [13–15], invertebrates [16, 17], economic plants [18, 19], alga [20] and actinomycetes [21]. In contrast to genomic sequence resources, the BAC libraries provide natural DNA materials for a variety of experiments, such as intact gene cluster cloning, map-based cloning, whole genome sequencing, comparative genomics analysis and fluorescence in situ hybridization that aim to understand functional elements in the genome [22, 23]. With the rapid development of high-throughput sequencing platforms like Illumina, PacBio and Oxford Nanopore, a high-quality, chromosome-level genome assembly could be effortlessly performed within acceptable prices in a short time [24]. Some spefical regions like telomere, centromere, rDNA, Y chromosomes have became the last bastion for generating gap-free and telomere-to-telomere chromosomes [25]. The utility of the BAC libraries can be greatly enhanced by mapping the BAC clones on the genome assemblies. However, the connection between assemblies and BAC libraries is still laborious and unaffordable [26].

Hence, we constructed a high-quality BAC library for broomcorn millet, developed a new pipeline for cost-effectively decoding BAC end seqeunces generated by clone-array pooled shotgun sequencing strategy (abbr. CAPSS) and Illumina sequencing technology, and mapped the BAC clones on the genome assemblies of the broomcorn millet [27].

## Results

### BAC library construction

**Fig. 1:**
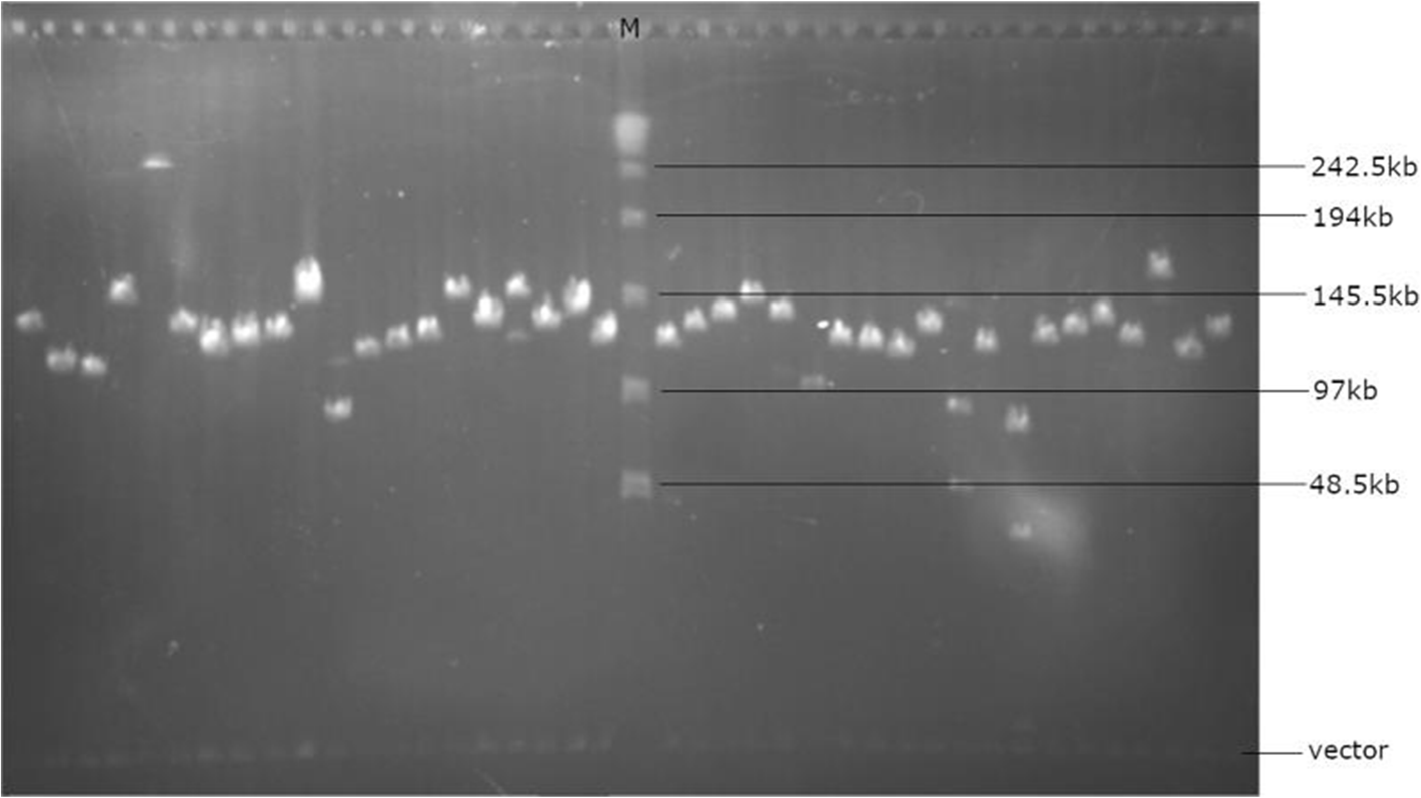
The insert sizes of randomly selected BAC clones determined by PFGE. The maker in the middle is *λ* DNA ladder

A BAC library of broomcorn millet was constructed with the restriction enzyme *Hin*dIII using high-molecular-weight genomic DNA prepared from etiolated seedlings. In total, the library consists of 76,032 BAC clones, that were arrayed into 198 independent 384-well microtiter plates. Insert sizing of randomly picked clones showed that the majority of genomic BAC inserts fell into the length range of 97–145.5 kb with an average insert size of 132 kb (Fig. 1).

### Construction and sequencing of DNA pools

**Fig. 2:**
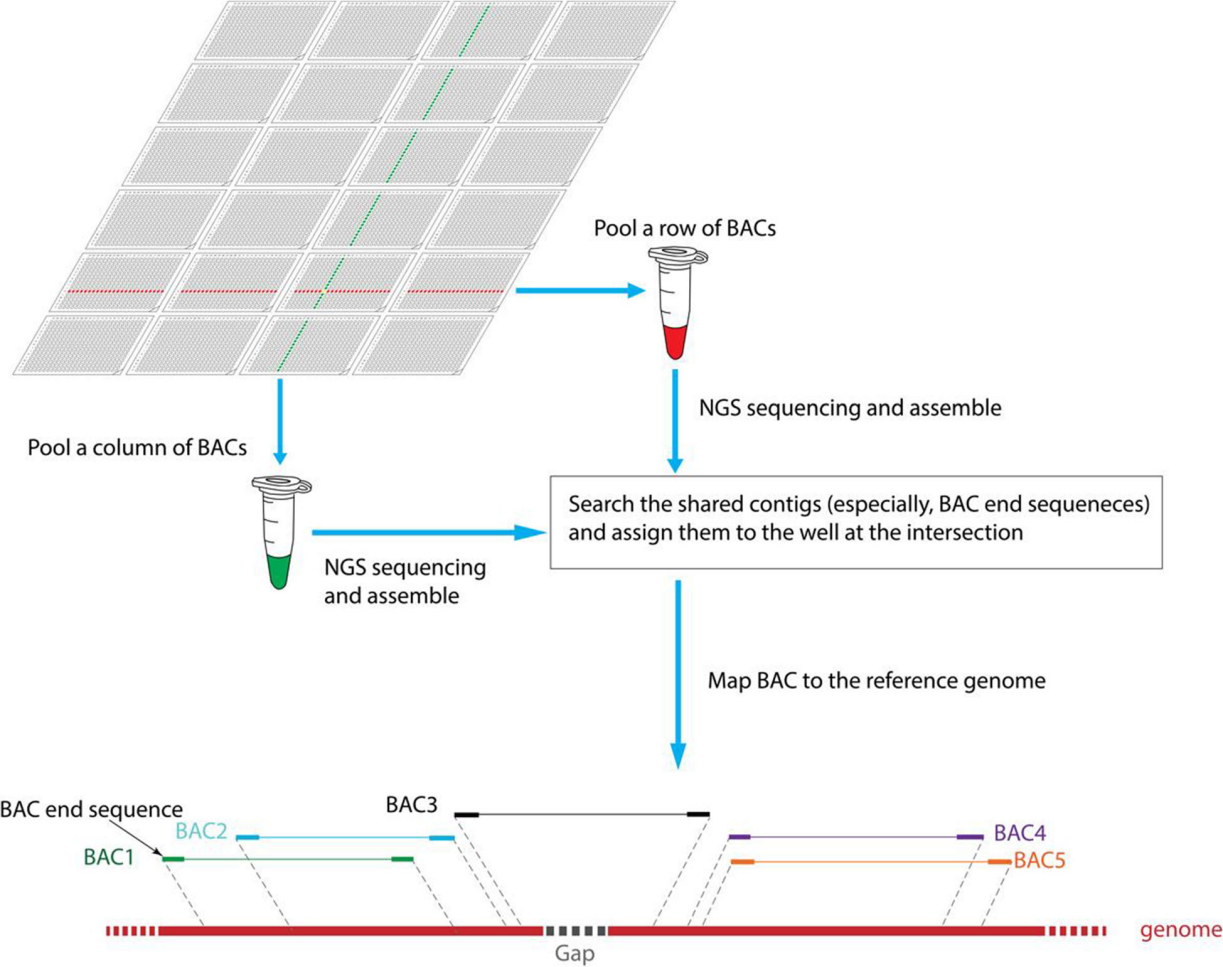
The strategy of BAC end sequence localization based on CAPSS. Twenty-four 384-plates are arranged to a square superpool, and then 96 row pools and 96 column pools are prepared and sequenced by NGS platform. The sequences of each pool are assembled into contigs. If a contig (especially, BAC end sequences, abbr. BES) is shared in a row pool and a column pool, it will be assigned to the well at the intersection of the row pool and the column pool. BAC clones will be further mapped to the reference genome according to assigned contigs

In order to obtain BAC end sequences with high efficiency and low cost, we designed and performed a pipeline based on the clone array pooled shotgun sequencing strategy (Fig. 2). Randomly selected 24, 384-plates (each plate consists of 16 × 24 clones) from the broomcorn millet BAC library were arranged to a square superpool with 6 row plates and 4 column plates. Therefore, the superpool consisted of 96 row pools (6 plates × 16) and 96 column pools (4 plates × 24). These row pools and column pools were called as the secondary pools, and each secondary pool also consisted of 96 BAC clones. In total, one superpool consisted of 192 secondary pools and 9216 individual BAC clones.

DNA of the secondary pools were extracted and Illumina sequencing libraries with individual index sequences for each secondary pool were prepared. Finally, 96 row pool libraries were mixed in equal amounts as the library X, and 96 column pool libraries were also mixed in equal amounts as the library Y. The average insert sizes of the library X and Y analyzed by the Agilent 2100 Bioanalyzer (Additional file 1: Fig. S1) were 491 bp and 443 bp, respectively. Sequencing was accomplished using the Illumina HiSeq 2000 sequencing platform with paired-end protocol (PE150). The Library X and Y generated 130.82 Gb and 133.50 Gb raw data, respectively. Trimmomatic was employed for trimming adaptors and filtering low-quality or shorter reads. FastQC was employed for evaluating the quality of the preprocessing reads. Then, the valid reads were obtained by filtering the reads traced to *E. coli* DH10B reference genome using Bowtie2 with default parameters. Finally, we obtained valid yields of 60.40 Gb and 102.37 Gb for libraries X and Y, respectively (Table 1). Demultiplexer was employed for demultiplexing and generating each secondary pool reads according to the 7-bp index (Additional file 2). The average valid sequencing depths of row pools and column pools were 45x and 78x, respectively (Additional file 1: Fig. S2).

**Table 1:** The summary of NGS data

Library	Total size (Gb)	Size after QC (Gb)	Valid size (Gb)
X	130.82	103.78	60.40
Y	133.50	107.92	102.37

### Parsing BAC end sequences

We focused our interest on the two special contigs in a BAC: the forward and reverse BAC end seqeunces. We designed two pathways to find them. On one hand, the paired-end reads overlapping with the border sequences of the vector harboring the *Hin*dIII restriction enzyme site were extracted from the valid data of each secondary pool, and then assembled using Cap3 based on the overlap-layout-consensus method. The vector sequence parts in the consensus were truncated to obtain short BAC end sequences (abbr. BES) starting with AAGCTT (*Hin*dIII site). These short BAC end sequences were assigned to the corresponding wells according to the clone array pooled shotgun sequencing strategy. Of the 9216 wells in 384-plates tested in the pipeline, 8183 (88.79%) wells were assigned one forward BES and 7897 (85.69%) wells were assigned one reverse BES (Additional file 3: Table S1). All assigned BAC end sequences have an average length of 358 bp (Additional file 1: Fig. S3), which is consistent with the insert size of the Illumina libraries (Additional file 1: Fig. S1).

On the other hand, the valid NGS data of each secondary pool were firstly assembled using SPAdes, and the contig N50 sizes of all row pools and column pools were counted (Additional file 1: Fig. S4). The average N50 of row pools and column pools were 13.97 kb and 11.45 kb, respectively. Likewise, the vector sequence parts at the ends of the contigs were removed to retain the long BESs starting with AAGCTT. Finally, these long BESs were assigned to the corresponding wells in 384-plates. Of the 9216 wells, 5454 (59.18%) wells obtained one forward BES and 5108 (55.43%) wells obtained one reverse BES (Additional file 3: Table S1). The N50 of the long BESs is 60.87 kb (Additional file 1: Fig. S5).

### Determination of BAC locations on the genome

In order to determine the locations of the broomcorn millet BAC clones on the genome, we aligned the short and long BAC end sequences onto the cultivar longmi4 genome with Blastn. Table 2 listed the alignment results. With BAC end sequences with repeats, 14,862 (83.44%) short BAC end sequences including 12,971 single-hit and 1891 multi-hit sequences were mapped to the genome, and 12,120 (95.28%) long BAC end sequences (all single-hit) were mapped to the genome. With the BAC end sequences without repeats, 7760 (43.57%) short BAC end sequences including 7295 single-hit and 465 multi-hit sequences were mapped to the genome, and 10,626 (83.53%) long BAC end sequences (all single-hit) were mapped to the genome. In order to map as many as possible BACs to the genome, the alignment results of the end sequences with repeats were adopted. We wrote a python script to extract the Blast results from short and long BESs. As a result, 5795 BACs were mapped to the genome using short BAC end sequences, and 6973 BACs were mapped to the genome using long BAC end sequences. Finally, the Blast results generated by short and long BAC end sequences were integrated, and in total 8262 BACs (89.65%) were mapped to the genome.

**Table 2:** A summary of the broomcorn millet BESs and the anchoring results of the broomcorn millet BAC clones to the longmi 4 genome using the BESs

Categories	Short BESs	Long BESs	Integrity
BAC end sequences			
Clones in superpool	9216	9216	
Clones with successful BESs	9126	7725	
Clones with paired BESs	7790	3890	
Clones with single-end BESs	1336	3835	
Clones with only forward BES	921	2189	
Clones with only reverse BES	415	1646	
Total successful BESs	17,811 (96.63%)	12,721 (66.17%)	
Alignment			
Aligned BESs with repeats unmasked	14,862 (83.44%)	12,120 (95.28%)	
Single-hit BESs	12,971	12,120	
Multi-hit BESs	1891	0	
Aligned BESs with repeats masked	7760 (43.57%)	10,626 (83.53%)	
Single-hit BESs	7295	10,626	
Multi-hit BESs	465	0	
Anchoring to reference sequences			
Clones anchored to single sites	5795	6973	8262
Clones anchored with single BES	2507	3907	2871
Clones anchored with paired BESs	3288	3066	5391

To verify the accuracy of BAC locations on the genome, 55 BAC clones were randomly picked from 384-plates for BAC end sequencing using Sanger method. After quality control, the 55 paired Sanger end sequences were blasted with the above short and long BESs, and the genome. All BAC clones except one (32G16) were consistent. By checking the 32G16 BAC end sequences, we found that this clone (or rather 32G16 well) was assigned two long forward BESs, one short forward BES, and no reverse BES. Only one long forward BES and the short forward BES were perfectly identical to the Sanger end sequence. The restriction of integration for the final mapping result has been adjusted to conquer this situation according to the consistency between long BESs and short BESs. In summary, the accuracy of the BAC mapping approach in this study was extremely high.

**Fig. 3:**
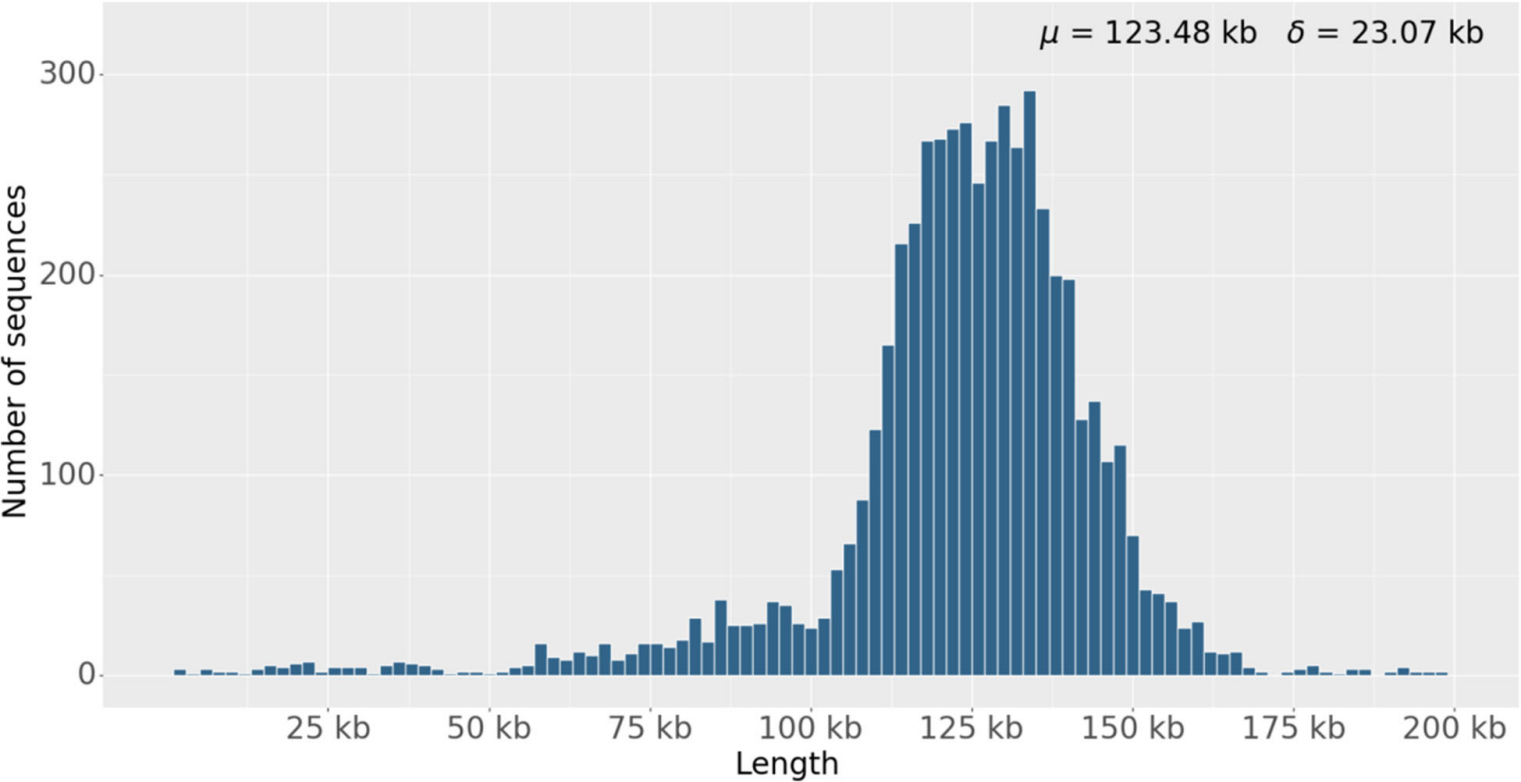
The statistics of the BAC inserts mapped by paired BESs

The distribution of the 5391 BACs mapped to the genome by paired BAC end sequences were counted (Table 3). These clones covered a total of 432.47 Mb of chromosomes with a total coverage of 50.97%. Among the 18 chromosome sequences of the cultivar longmi4 genome, 829 gaps were left. Our BACs covered 308 of them. The insert sizes of these BACs presented the Gaussian distribution, with an average insert size of 123.48 kb (Fig. 3), which is lower than that predicted by pulse field electrophoresis. These BACs are valuable resources for further improvement of the genome.

**Table 3:** The location result of BACs on broomcorn millet chromosomes

Chr.	Length (Mb)	No. of gaps	No. of BACs	BACs Covered	
				Length (Mb)	No. of gaps
chr 1	69.18	85	439	35.25	34
chr 2	61.15	59	379	31.04	19
chr 3	57.97	50	394	31.03	20
chr 4	56.29	34	359	27.90	14
chr 5	54.13	52	361	29.86	19
chr 6	52.84	46	358	28.24	16
chr 7	51.23	67	286	23.67	27
chr 8	48.26	29	339	26.49	8
chr 9	45.11	70	240	20.96	21
chr10	44.65	53	308	25.44	28
chr11	43.18	30	267	21.90	14
chr12	42.47	30	254	20.79	11
chr13	40.72	50	261	21.11	17
chr14	38.49	32	269	20.23	10
chr15	34.36	34	213	18.14	8
chr16	33.61	45	212	16.72	15
chr17	32.99	25	226	18.26	10
chr18	32.24	38	199	15.32	17
unplaced	9.48	0	27	–	0
Total	848.47	829	5391	432.47	308

### Presentation of BAC locations by JBrowse

**Fig. 4:**
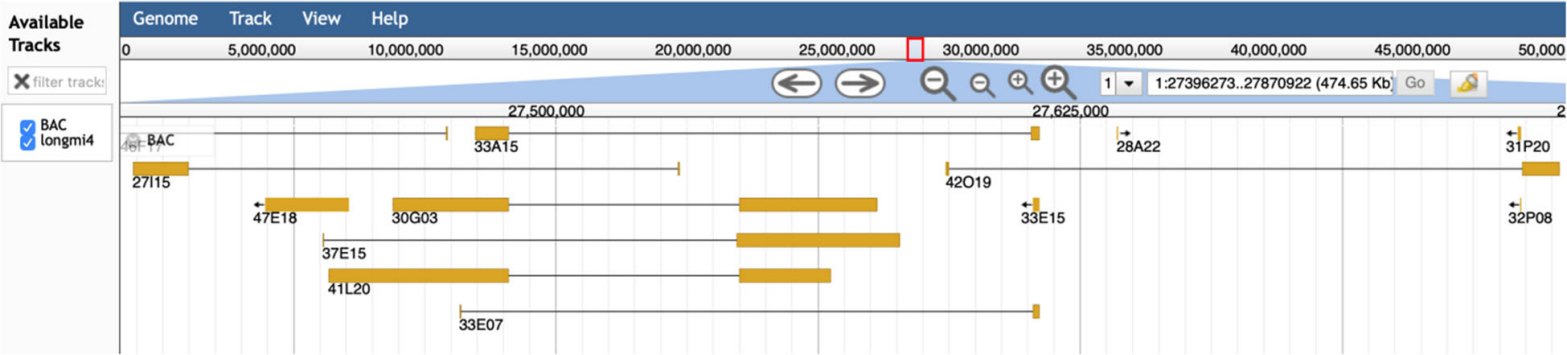
Representation of broomcorn millet BAC locations by JBrowse. A barbell icon idicates a BAC mapped by forward and reverse BESs (yellow rectangle). An icon with arrow indicates a BAC mapped by only single BES

In order to effectively use the BAC resource, quickly and easily retrieve BAC clones and analyze other annotation information, we established a resource website employing a lightweight JBrowse to display the BAC resource information on the broomcorn millet genome (Fig. 4).

### Organelle genomes of the broomcorn millet

We aligned all BESs in this study to the chloroplast (CM009689) genome of broomcorn millet by local blast. A total of 65 BAC clones were mapped to the chloroplast with more than 99% of similarity, of which 16 BAC clones were determined by paired BESs (25C07, 25 N09, 27F16, 27O19, 29B02, 29 K11, 32 J09, 32 N16, 33D23, 33 K24, 36A05, 39C22, 42C10, 46C16, 48B15, 48C03). In the absence of mitochrondrial sequence in broomcorn millet, we aligned BESs to all mitochondrial genomes in *Gramineae*. Of 16 BAC clones with homologous BES sequences, 13 BACs were simultaneously aligned to the chloroplast and 3 BACs were simultaneously aligned to the nuclear genome.

## Discussion

BAC library is still a powerful resource for genome assembly, functional genomics research, and long-term genetic resoure storage for the endangered species. BAC seqeunces, espcially paired BESs, are usally used to detect assembly errors or assist assembly [28]. The most conventional and convenient approach to decode BESs is Sanger sequencing method. However, its one-by-one style is laborious, time-consuming and expensive. Fortunately, a few of efficient approaches had developped, based on next generation sequencing technologies, such as pBACcode and BAC-anchor [26, 29]. pBACode determines paired BESs by a pair of random barcodes flanking the cloning site. BAC-anchor determines general paired BESs by using specific restriction enzyme sites and searching for utlra-long paired-end subreads containing large internal gaps. We previously also developed a high-throughput approach for long accurate BES profiling with PacBio sequencing technology [30]. However, these approaches focused on paired BES profiles, and they are hard to trace BESs of specific clones in 384-plate wells. In this study, we generated BESs and assigned them to physical wells in 384-plates by applying the characteristics of row-cross-column in two-dimensional arrays and cost-effective Illumina platform. These BAC end sequences in average are longer and accurate than that generated by Sanger method, for assembling by SPAdes assembler [31]. After alignment, 89.65% of clones were successfully associated with the broomcorn millet longmi 4 genome. These clones covered 308 of the 829 gaps left by the genome and can be uesd to close the genome gaps.

In conventional whole genome sequencing project, sequencing coverage is usallly at least 30x, and not more than 200x. Higher coverage will result in more sequences that are generated by PCR mutations or sequencing errors and lead to contigs with shorter N50. We added index into each secondary pool and mixed 96 secondary pools as a sequencing library to run in a lane of Illumina flow cell for greatly reducing the cost of sequencing. As a result, after removing the contaminated *E. coli* genomic DNA reads the average valid sequencing depths of row pools and column pools were 45x and 78x, respectively.

In the process of BESs extraction, we designed two pathways: short BES pathway and long BES pathway. Short BES pathway searched all reads overhanging with verctor end sequences before assembling by Cap3; consequently, it generated all potenital BESs. Long BES pathway identified all contigs overhanging with vector end seqeunces after assembling by SPAdes; consequently, it generated longer but less BESs than the first pathway. The assignment of BAC end sequences at the intersection sites is affected by many factors, such as the overlapping rate of the BAC clones and the correct rate of the sequences. The overlapping rate of BAC clones in the superpool is the most important factor. If overlapping BAC clones appear in the same row or column pool, we can assign them to wells easily and correctly. Also, if two overlapping BAC clones appear in a different row or column pool, we can rectify them by our previous method [32]. However, if more than two overlapping BAC clones appear in a different row or column pool, our method will filter out potential BESs, so that the BESs of the intersection well will be absent. In the process of BES assignment, the flow of the forward and reverse BESs are completely independent. When more than one forward and/or reverse BESs were assigned to a well, we cannot determine which BESs are a pair of BESs. If a high-quality genome is available, it is easy to assess which pair of BESs are derived from the same BAC by mapping. However, if the variety used for BAC library construction is not the same as that the reference genome stands for, the alignment results that do not satisfy the location requirement of BES pairs will be discarded. The clones that may contain a large structural variation can be picked out for further analysis from the 384-wells plate.

Plant cells contain an abundance of chloroplasts and mitochondria. The chloroplast genome is generally around 150 kb, while the mitochondrial genome size varies widely, typically between 200 kb and 750 kb [33]. Although nuclei are extracted for BAC library construction, a trace of organelle DNA cotamination is inevitable. In this study, a small number of clones of chloroplast genome was found by BESs, while clones of mitochondrial genome were almost absent.

By high-throughput sequencing and mapping clones in the secondary pool of BAC libraries, we can find the coordinates of genome sequences in the broomcorn millet BAC libraries. Therefore, if we find genes that play an important role in biology, we can quickly locate the BAC clones containing this gene, and obtain the experimental materials for further research and analysis. At the same time, because BAC contains a long DNA fragment (about 120 kb), it is also convenient for us to quickly analyze the upstream and/or downstream DNA elements of interested genes or adjacent genes.

## Conclusions

We constructed a high-quality BAC library for broomcorn millet, developed a high-efficient and low-cost pipeline which can generate and parse ten thousand of paired BAC end sequences at each time, and mapped a total of 8262 broomcorn millet BACs to the chromosomes. These clones covered 308 of the 829 gaps left by the genome. The high-quality BAC clones mapped on genome in this study will provide a powerful genomic resource for gap filling, complex segment sequencing, FISH, functional research, and genetic engineering of broomcorn millet.

## Methods

### Plant materials, growth conditions and BAC library construction

The broomcorn millet landrace was provided by professor Mingsheng Chen of the Institute of Genetics and Developmental Biology, Chinese Academy of Sciences. Plants were grown at dark conditions under 25 °C. The young leaves of seedlings were harvested and mixed, frozen immediately in liquid nitrogen for the extraction of nuclear DNA. High molecular weight nuclear DNA was extracted and BAC library was constructed following our previous protocol [10]. Partial digestions of DNA plugs with dilution *Hin*dIII were performed. DNA fragments ranging from 100 kb to 200 kb were recovered from pulse field gel and ligated with pIndigoBAC536-S vector [11]. The ligation product was used to transform DH10B cells by electroporation. White colonies were picked up and stored in 384-plates at − 80 °C.

### Pool construction

Twenty-four 384-plates were chosen from the BAC library of broomcorn millet. These plates were arranged in a 2-dimension superpool. The superpool contains 96 row pools and 96 column pools. A total of 192 pools were processed independently for high quality DNA extraction with the AxyGen AxyPrep Easy-96 plasmid kit. Then, these BAC DNAs were completely digested with ATP-Dependent DNase (Epicentre) to remove the host *E. coli* DNA. After digestion, these BAC DNAs were sheared in the Bioruptor to an average of 500 bp. During blunt-end repair, overhanging 5′ and 3′ ends were filled in or removed by T4 DNA polymerase. 5′-phosphates were attached using T4 polynucleotide kinase. Tail-A were added using Taq DNA polymerase. Then, adapters were ligated to both ends of the molecules using T4 DNA ligase. The ligation products were cleaned using MagBead DNA Purification Kit (Sangon Biotech, shanghai, CN). Sequencing adaptors were added using PCR. Finally, the products of KOD PCR were cleaned again.

### Illumina sequencing and BAC end sequence analysis

NGS sequencing of 2 mixed DNA libraries was performed via the Illumina HiSeq 2000 with 150-bp paired-end protocol (Genewiz, suzhou, CN). Trimmotatic was employed to filter and trim raw reads. FastQC was employed to assess data quality. After adapter filtering and quality assessment, BBMAP/demuxbyname script was employed for deconvolution depending on unique index sequence in each pool. A python script was employed to extract target paired-end reads that cover BES-VES site. Cap3 was employed to assemble those reads to consensuses, and consensuses were then trimmed to remove the part of vector to generate BESs called “short BES”. SPAdes was employed to directly assemble pool reads to contigs. Then, the contigs containing BAC vector sequences were extracted and trimmed using python script to generate the trimmed contigs called “long BES”. Blastn was used to align BESs from row and column pools, and then the shared BESs were assigned to the wells at the intersection.

### Validation of BAC end sequences

The analysis results of the BAC end sequences were validated by Sanger sequencing. Fifty-five BAC clones were randomly selected and their DNAs were extracted using an improved alkaline lysis protocol. Sanger sequencing was accomplished using BAC-F (5′-AACGACGGCCAGTGAATTG-3′) and BAC-R (5′-GATAACAATTTCACACAGG-3′) primers from pIndigoBAC536-S vector backbone. The BAC end sequences from Sanger sequencing were aligned to BES from the analysis results of illumina data using Blastn.

### BAC mapping on broomcorn millet longmi4 genome

The genome sequence of broomcorn millet longmi4 was downloaded from NCBI genome database under GCA_002895445 accession, which was submitted by researchers from China Agricultural University. The local blastn was employed to map all BESs to the genome with the following options: qcov_hsp_perc = 99, perc_identity = 99, outfmt = 6, culling_limit = 1. The results were further converted to a GFF3 format file using a Python script. In this script, following conditions were set: if both forward and reverse BESs in each clone were mapped to the same chromosome, and their orientations were opposite and their interval lengths were less than 250 kb, such clones were recorded in GFF3 format file with three lines; if either forward or reverse BES was uniquely mapped to chromosome, such clones also were recorded in GFF3 format file with two lines; other conditions would be discarded. The GFF3 file was sorted using GFF3sort and presented with JBrowse in our website (http://eightstarsbio.com/gresource/JBrowse-1.16.5/index.html).

### Organelle genome analysis

The chloroplast genome (CM009689) of brromcorn millet and all mitochondrial genomes (NC_008331, NC_007982, NC_036024, NC_031164, NC_029816, NC_022714, NC_022666, NC_013816, NC_007886, NC_011033, NC_008362, NC_008360, NC_008332, NC_008333) in *Gramineae* were downloaded from NCBI nucleotide database. All potential BACs of organelle genome were identified by the local blastn with following options: qcov_hsp_perc = 99, perc_identity = 99, outfmt = 6.

## Supplementary Information

**Additional file 1: Fig. S1.** Distribution of DNA fragment lengths in library X and Y analyzed by the Agilent 2100Bioanalyzer. The peak in the middle of each panel is the length range of DNA library. The peaks on the left andright of each panel are 25 bp and 1500 bp DNA standards, respectively. (A) Sequencing library X, (B) Sequencing library Y. **Fig. S2.** Distribution of the coverage numbers of valid reads in pools of sequencing library X and Y. Theaverage BAC insert size estimated by PFGE is 132 kb and the length of pIndigoBAC536-S vector is 7 kb, so thecoverage number of valid reads in each pool is calculated with an average BAC size of 139 kb. Fig. S3.Distribution of short BAC end sequence lengths. **Fig. S4.** Distribution of the lengths of contig N50 assembled by SPAdes in pools of sequencing library X and Y. **Fig. S5.** Distribution of long BAC end sequence lengths.

**Additional file 2.** The index information of each secondary pool.

**Additional file 3: Table S1.** the number of wells assigned with BESs.

## Data Availability

The source codes are openly available in a GitHub repository (https://github.com/xuweixw/broomcorn-millet-BAC-library). Illumina sequencing data are available at Sequence Read Archive (SRA) under the accession PRJNA576359. BAC clones can be browsed and obtained through our website (http://eightstarsbio.com/gresource/JBrowse-1.16.5/index.html).

## Abbreviations

BAC: bacterial artificial chromosome
BES: BAC end sequence
CAPSS: clone array pooled shotgun sequencing
CM: chloramphenicol
NGS: next generation sequencing
PCR: polymerase chain reaction
VES: vector end sequence
QTL: quantitative trait locus

## References

[1] Kalinova J, Moudry J (2006). Content and quality of protein in proso millet (Panicum miliaceum L.) varieties. Plant Foods Hum Nutr.

[2] Washburn JD, Schnable JC, Davidse G, Pires JC (2015). Phylogeny and photosynthesis of the grass tribe Paniceae. Am J Bot.

[3] Baltensperger DD. Progress with proso, pearl and other millets. In Trends in New Crops and New Uses. Alexandria: ASHSPress; 2002.

[4] Dong YC, Liu X. Crops and their wild relatives in China. Beijing: China Agriculture Press; 2006.

[5] Liu M, Xu Y, He J, Zhang S, Wang Y, Lu P (2016). Genetic Diversity and Population Structure of Broomcorn Millet (Panicum miliaceum L.) Cultivars and Landraces in China Based on Microsatellite Markers. Int J Mol Sci.

[6] Zhang X, Guan Z, Wang L, Fu J, Zhang Y, Li Z, Ma L, Liu P, Zhang Y, Liu M, Li P, Zou C, He Y, Lin H, Yuan G, Gao S, Pan G, Shen Y (2020). Combined GWAS and QTL analysis for dissecting the genetic architecture of kernel test weight in maize. Mol Gen Genomics.

[7] Humira S, Louise O, Elroy C, Istvan R, François B (2015). Identification of loci governing eight agronomic traits using a GBS-GWAS approach and validation by QTL mapping in soya bean. Plant Biotechnol J.

[8] Shi J, Ma X, Zhang J, Zhou Y, Liu M, Huang L, Sun S, Zhang X, Gao X, Zhan W, Li P, Wang L, Lu P, Zhao H, Song W, Lai J (2019). Chromosome conformation capture resolved near complete genome assembly of broomcorn millet. Nat Commun.

[9] Zou C, Li L, Miki D, Li D, Tang Q, Xiao L, Rajput S, Deng P, Peng L, Jia W, Huang R, Zhang M, Sun Y, Hu J, Fu X, Schnable PS, Chang Y, Li F, Zhang H, Feng B, Zhu X, Liu R, Schnable JC, Zhu JK, Zhang H (2019). The genome of broomcorn millet. Nat Commun.

[10] Luo M, Wing RA. An improved method for plant BAC library construction. Methods Mol Biol. 2003;236:3–20. 10.1385/1-59259-413-1:3. 10.1385/1-59259-413-1:3 14501055

[11] Shi X, Zeng H, Xue Y, Luo M (2011). A pair of new BAC and BIBAC vectors that facilitate BAC/BIBAC library construction and intact large genomic DNA insert exchange. Plant Methods.

[12] Shizuya H, Birren B, Kim UJ, Mancino V, Slepak T, Tachiiri Y, Simon M (1992). Cloning and stable maintenance of 300-kilobase-pair fragments of human DNA in Escherichia coli using an F-factor-based vector. Proc Natl Acad Sci U S A.

[13] Zeng CJ, Pan HJ (2007). Gong S bin, Yu JQ, wan QH, fang SG. Giant panda BAC library construction and assembly of a 650-kb contig spanning major histocompatibility complex class II region. BMC Genomics.

[14] Asakawa S, Abe I, Kudoh Y, Kishi N, Wang Y, Kubota R, Kudoh J, Kawasaki K, Minoshima S, Shimizu N (1997). Human BAC library: construction and rapid screening. Gene.

[15] Xu H, Qian Y, Nie W, Chi J, Yang F, Su B (2004). Construction, characterization and chromosomal mapping of bacterial artificial chromosome (BAC) library of Yunnan snub-nosed monkey (Rhinopithecus bieti). Chromosom Res.

[16] Ma K, Yu S, Du Y, Feng S, Qiu L, Ke D (2019). Construction of a genomic bacterial artificial chromosome (BAC) library for the prawn Macrobrachium rosenbergii and initial analysis of ZW chromosome-derived BAC inserts. Mar Biotechnol.

[17] Song X, Goicoechea JL, Ammiraju JSS, Luo M, He R, Lin J, Lee SJ, Sisneros N, Watts T, Kudrna DA, Golser W, Ashley E, Collura K, Braidotti M, Yu Y, Matzkin LM, McAllister BF, Markow TA, Wing RA (2011). The 19 genomes of Drosophila: a BAC library resource for genus-wide and genome-scale comparative evolutionary research. Genetics.

[18] Ammiraju JSS, Song X, Luo M, Sisneros N, Angelova A, Kudrna D, Kim HR, Yu Y, Goicoechea JL, Lorieux M, Kurata N, Brar D, Ware D, Jackson S, Wing RA (2010). The Oryza BAC resource: a genus-wide and genome scale tool for exploring rice genome evolution and leveraging useful genetic diversity from wild relatives. Breed Sci.

[19] Tai Y, Wang H, Wei C, Su L, Li M, Huang B (2017). Construction and characterization of a bacterial artificial chromosome library for Camellia sinensis. Tree Genet Genomes.

[20] Deng DY, Zhao G, Xuan JS, Yang JL, Duan DL, Weng ML (2004). Construction and characterization of a bacterial artificial chromosome library of marine macroalga Porphyrayezoensis (Rhodophyta). Genet Res.

[21] Deng Q, Li Z, Luo M, Deng Z, Zhao C (2017). Heterologous expression of Avermectins biosynthetic gene cluster by construction of a bacterial artificial chromosome library of the producers. Synth Syst Biotechnol.

[22] Pan Y, Deng Y, Lin H, Kudrna DA, Wing RA, Li L, Zhang Q, Luo M (2014). Comparative BAC-based physical mapping of Oryza sativa ssp. indica var. 93-11 and evaluation of the two rice reference sequence assemblies. Plant J.

[23] Dong G, Shen J, Zhang Q, Wang J, Yu Q, Ming R (2018). Development and Applications of Chromosome-Specific Cytogenetic BAC-FISH Probes in S spontaneum. Front Plant Sci.

[24] Shendure J, Balasubramanian S, Church GM, Gilbert W, Rogers J, Schloss JA, Waterston RH (2017). DNA sequencing at 40: past, present and future. Nature.

[25] Song J, Xie W-Z, Wang S, Guo Y-X, Koo D, Kudrna D (2021). Two gap-free reference genomes and a global view of the centromere architecture in rice. Mol Plant.

[26] Wei X, Xu Z, Wang G, Hou J, Ma X, Liu H, Liu J, Chen B, Luo M, Xie B, Li R, Ruan J, Liu X (2017). PBACode: a random-barcode-based high-throughput approach for BAC paired-end sequencing and physical clone mapping. Nucleic Acids Res.

[27] Cai WW, Chen R, Gibbs RA, Bradley A (2001). A clone-array pooled shotgun strategy for sequencing large genomes. Genome Res.

[28] Deng Y, Pan Y, Luo M (2014). Detection and correction of assembly errors of rice Nipponbare reference sequence. Plant Biol.

[29] Yang X, Yang Y, Ling J, Guan J, Guo X, Dong D, Jin L, Huang S, Liu J, Li G (2019). A high-throughput BAC end analysis protocol ( BAC -anchor) for profiling genome assembly and physical mapping. Plant Biotechnol J.

[30] Zhaozhao D, Tong L, Jiadong L, Zhifei H (2019). High-throughput long paired-end sequencing of a Fosmid library by Pacbio. Plant Methods.

[31] Bankevich A, Nurk S, Antipov D, Gurevich AA, Dvorkin M, Kulikov AS, Lesin VM, Nikolenko SI, Pham S, Prjibelski AD, Pyshkin AV, Sirotkin AV, Vyahhi N, Tesler G, Alekseyev MA, Pevzner PA (2012). SPAdes: a new genome assembly algorithm and its applications to single-cell sequencing. J Comput Biol.

[32] Pan Y, Wang X, Liu L, Wang H, Luo M (2016). Whole genome mapping with feature sets from high-throughput sequencing data. PLoS One.

[33] Gualberto JM, Newton KJ (2017). Plant mitochondrial genomes: dynamics and mechanisms of mutation. Annu Rev Plant Biol.

