# Supplementary material for: Genomic resources of broomcorn millet: demonstration and application of a high-throughput BAC mapping pipeline
Source: BMC Genom Data. 2021 Nov 1;22:46. doi: 10.1186/s12863-021-01003-z (PMC8561967; doi:10.1186/s12863-021-01003-z)
Supplement: Supplementary file 3 — Additional file 3: Table S1. the number of wells assigned with BESs. [file 12863_2021_1003_MOESM3_ESM.docx]

Genomic Resources of Broomcorn Millet: Demonstration and Application of a High-throughput BAC Mapping Pipeline

M.S. Wei Xu, M.S. Mengjie Liang, M.S. Xue Yang, Dr. Hao Wang, Prof. Meizhong Luo

Table S1 the number of wells assigned with BESs

| The Number of BESs | In short BES pathway | | In long BESs pathway | |
| --- | --- | --- | --- | --- |
|  | Forward | Reverse | Forward | Reverse |
| 0 | 505 | 1011 | 3138 | 3680 |
| 1 | 8183 | 7897 | 5454 | 5108 |
| 2 | 487 | 302 | 591 | 410 |
| 3 | 39 | 6 | 31 | 17 |
| 4 | 2 | - | 2 | 1 |
